# Access and Financial Burden for Patients Seeking Essential Surgical Care in Pakistan

**DOI:** 10.5334/aogh.3784

**Published:** 2022-12-20

**Authors:** Lubna Samad, Muhammad Nabeel Ashraf, Ammar Ali Mohammad, Irum Fatima, Zachary Fowler, Katherine Albutt, Asad Latif, John G. Meara, Manon Pigeolet

**Affiliations:** 1 Interactive Research and Development (IRD), Karachi, Pakistan; 2 Indus Hospital and Health Network, Pakistan; 3 Augusta University - Medical College of Georgia, Augusta, GA, USA; 4 Program in Global Surgery and Social Change, Harvard Medical School, Boston, MA, USA; 5 Zucker School of Medicine at Hofstra/Northwell, New Hyde Park, NY, USA; 6 Division of Trauma, Emergency Surgery and Surgical Critical Care, Massachusetts General Hospital, Boston, MA, USA; 7 Department of Anaesthesia, Aga Khan University, Karachi, Pakistan; 8 Department of Plastic and Oral Surgery, Boston Children’s Hospital, Boston, MA, USA; 9 Université Libre de Bruxelles, Faculty of Medicine, Brussels, Belgium

**Keywords:** Global Surgery, health financing, global health

## Abstract

**Background:**

Pakistan is a lower middle-income country in South Asia with a population of over 220 million. With the recent development of national health programs focusing on surgical care, two areas of high priority for research and policy are access and financial risk protection related to surgery. This is the first study in Pakistan to nationally assess geographic access and expenditures for patients undergoing surgery.

**Methods:**

This is a cross-sectional study of patients undergoing laparotomy, cesarean section, and surgical management of a fracture at public tertiary care hospitals across the country. A validated financial risk protection tool was adapted for our study to collect data on the socio-economic characteristics of patients, geographic access, and out-of-pocket expenditure.

**Results:**

A total of 526 patients were surveyed at 13 public hospitals. 73.8% of patients had 2-hour access to the facility where they underwent their respective surgical procedures. A majority (53%) of patients were poor at baseline, and 79.5% and 70.3% of patients experienced catastrophic health expenditure and impoverishing health expenditure, respectively.

**Discussion:**

A substantial number of patients face long travel times to access essential surgical care and face a high percentage of impoverishing health expenditure and catastrophic health expenditure during this process. This study provides valuable baseline data to health policymakers for reform efforts that are underway.

**Conclusions:**

Strengthening surgical infrastructure and services in the existing network of public sector first-level facilities has the potential to dramatically improve emergency and essential surgical care across the country.

## Introduction

Pakistan is a South Asian lower-middle-income country with an ethnically diverse population of over 220 million [1]. It has one of the most decentralized healthcare systems in the world, with nearly all of the decision-making being done on the provincial level [2]. Current governmental healthcare expenditure stands at only 1.5% of the Gross Domestic Product [3]. Surgical care is considered to be a specialized service that is complex and expensive and hence has been largely omitted from health agendas and initiatives in lower and middle-income countries (LMICs) [1,4,5]. Pakistan is not an exception to this rule despite modeled estimates of 187 deaths per 100 000 people per year from acute surgical conditions.

Pakistan is a vast [1], geographically diverse country with underdeveloped transportation infrastructure. This situation leads to significant challenges in access to health facilities since the few tertiary care facilities providing the bulk of surgical services are located in large urban centers. Travel times to access care can therefore be very long, resulting in compromised surgical care, especially in rural and distant parts of the country. In order to allow for the development of health policies and programs that address this reality, a thorough understanding of access challenges faced by patients is necessary.

Compounding the difficulties in geographical access, costs related to surgical care can pose insurmountable barriers to patients in Pakistan. In the absence of adequate financing and insurance schemes, surgery can impose severe financial hardship on patients and their households. Impoverishing Health Expenditure (IHE) and Catastrophic Health Expenditure (CHE) are measures quantifying the financial burden put on people when seeking care in a certain country or region [6]. Current literature has looked predominantly into the relationship between IHE/CHE and surgery in sub-Saharan Africa, showing that poor patients are at very high risk of experiencing CHE or IHE when seeking surgical care. Data from Asia is largely absent in literature [7], and this remains an area that needs to be studied beyond the African continent.

The Lancet Commission on Global Surgery recommended six core indicators to measure the accessibility, availability and safety of surgical care, including indicators looking into 2-hour access and financial risk protection as measured by CHE and IHE [4]. Robust and nationally representative data on surgical care, including the aforementioned surgical indicator data was unavailable for Pakistan. The importance of systematic data on surgical care to understanding the true state of surgical services has been emphasized by the Lancet Commission as an essential step in understanding and subsequently addressing gaps in surgical care delivery [4]. This study aims to assess access to and utilization of surgical care, as well as impoverishing and catastrophic health expenditure (IHE and CHE, respectively) as a result of seeking surgical care at public tertiary care hospitals in each province of Pakistan.

## Methods

### Study design

This cross-sectional study was conducted from May 2019 to January 2020. To ensure selection of high volume and geographically diverse facilities, a systematic approach was used to select 13 tertiary public sector hospitals across the country: two from Balochistan, one from Islamabad Capital Territory (ICT), three each from Khyber Pakhtunkhwa (KP) and Punjab, and four from Sindh (Table 1). Using an estimated proportion of CHE of 0.5, a 95% confidence interval, and a margin of error (alpha) of 0.043, a sample size of 520 was needed. Using a volume threshold basis, approximately 40 patients were surveyed from each hospital to reach a total sample size of 526 patients.

**Table 1:** List of facilities.


PROVINCE	HOSPITAL

Balochistan	Bolan Medical College Hospital, Quetta

Balochistan	Civil Hospital Sandeman, Quetta

ICT	Pakistan Institute of Medical Sciences, Islamabad

KPK	Ayub Medical Teaching Hospital, Abbottabad

KPK	Khyber Teaching Hospital, Peshawar

KPK	Swat General Teaching Hospital, Mingora

Punjab	Allied Hospital and DHQ Hospital, Faisalabad

Punjab	Mayo General Hospital & Lady Willingdon Hospital, Lahore

Punjab	Nishtar Hospital, Multan

Sindh	Civil Hospital Hyderabad, Hyderabad

Sindh	Ruth Phao Civil Hospital, Karachi

Sindh	Chandka Medical College Hospital, Larkana

Sindh	Ghulam Mehr Medical College, Sukkur


### Participants

Post-operative patients who had undergone a Cesarean section (C-section), laparotomy or surgical management of a fracture at these hospitals were included in the study. These are termed as Bellwether procedures, except we adapted our definition of fractures for the purpose of our study, including surgical management of all fractures. This was due to the fact that patient understanding of open versus closed fracture is often limited in our population. In order to ensure adequate representation of all three procedures, C-section enrollments were limited to 50% of participants at each hospital. Patients who underwent procedures other than these three interventions or were waiting for their procedures were not included. There was no age requirement for inclusion or exclusion in the study.

### Interview instrument

The interview tool included questions from a validated patient survey on Financial Risk Protection (FRP) developed by the Harvard Program in Global Surgery and Social Change [7,8]. This tool collects information about average monthly income, monthly household expenditures, and the out-of-pocket expenditures (OOP) incurred in seeking surgical care. The adapted tool is included in Appendix 1. Information was also collected on socioeconomic status, accessibility, expenditures, and hardship financing to elicit a deeper understanding of the surgical experience. Patient-specific variables included the facility at which the patient was treated, the type of procedure performed on the patient, and the length of stay.

### Variables

Socioeconomic variables, including the patient’s age, sex, education, primary earner income and expenditures on health, transport, housing, clothing, food, livestock, education, household size, occupation of primary earner, residential address, and residence within or outside the city were collected. Primary earner occupation was categorized as informal (laborers, farmers, drivers), business owners/self-employed (which includes shopkeepers, small-scale business owners), private employees, government employees, and unemployed. Accessibility variables included categorical variables (type of transportation used, the availability of funds to arrange transportation, and access within 2 hours) and continuous variables (number of stops and time taken to arrange transportation and reach the hospital).

The expenditure variables included direct medical costs (doctor’s fees, medicine, bandages and dressings, laboratory costs, and radiology costs) and direct non-medical costs (patient and attendant travel, food, and accommodation costs). Hardship financing variables included binary variables for patients who took a loan, sold a possession, interrupted their children’s education, lost employment, or accepted charity.

Variables created after data collection included binary variables identifying patients who were living below the defined poverty line, reached the hospital within 2 hours of travel time, suffered CHE or IHE, or had to rely on any type of hardship financing [9]. Continuous variables denoting total direct expenditures, including medical and non-medical expenditures experienced by each patient were also created. Conversion from Pakistani rupees (PKR) to United States dollars (USD) was based on the purchasing power parity (PPP) conversion factor for private consumption in 2019 [10]. Poverty was defined using the World Bank definition of USD 3.2 (PPP) per capita per day— equivalent to PKR 118 per capita per day—and was based on household size and the aggregated monthly family consumption [11]. This threshold allows for comparison with international literature and is also similar to the Pakistan Institute of Development Economics’ defined national poverty threshold of USD 3.4 (PPP), equivalent to PKR 125.8 per capita per day [12]. IHE was defined as total health expenditure exceeding the difference between the patients’ per capita annual expenditure and the World Bank’s annual per capita poverty threshold. CHE was defined as expenditure exceeding 10% of patients’ total annual per capita expenditure [13,14].

### Data collection

Data collection was conducted over 1–2 days at each study site by two community health workers who had been trained to administer the tool. These health workers were supervised by the field supervisor, who reported to a program coordinator (a medical doctor) on a daily basis. The program coordinator conducted monitoring visits to ensure quality and consistency. After obtaining relevant permissions from the hospital administration, health workers would visit the surgical, obstetric, and orthopedic wards to enroll post-operative patients who were eligible for the study. Informed consent was obtained, and the interview with the patient or attendant was conducted. Data was collected on paper forms and later entered by the health workers into the secure REDcap online application hosted on the institutional server [15,16]. All paper and consent forms were transported by the program coordinator to the main office where they were stored in a secure locker. Names of participants were not recorded; instead, the patients were given codes to maintain the privacy and confidentiality of the data.

### Data analysis

STATA version 14 was used for analysis [17]. The data was checked for validity, completeness, consistency, and uniformity. Missing data was filled by checking the paper forms. Descriptive analysis was conducted for the demographic, access, and financial data. Continuous variables were summarized with either means and standard deviations (SD) or medians and interquartile ranges. Categorical variables were summarized as frequencies and percentages. Financial data analysis included summarizing the direct medical, non-medical, and total direct expenditures as well as the frequency of hardship financing, CHE, and IHE.

### Ethical approval

Ethical approval to conduct the study was received from the Interactive Research and Development Institutional Review Board (IRD IRB 2018 05 006) and the Harvard Medical School Institutional Review Board. The Ministry of National Health Service Regulation and Coordination, the Government of Pakistan, the provincial departments of health, and the selected hospitals issued letters of support to conduct the study.

## Results

### Socioeconomic characteristics

We surveyed 526 surgical patients at 13 facilities (Table 1). This included 171 patients recruited from Sindh, 114 from Punjab, 80 from Balochistan, 120 from KP, and 41 from ICT. Patients had undergone C-section (241), laparotomy (143), and surgical management of fracture (142). Most patients were female (58.6%); however, if C-sections were excluded, the majority of participants were male (76.5%). Females constituted only 17.6% and 29.4% of laparotomy and surgical management of fracture groups, respectively. The participant ages ranged from 3–90 years, with the majority (75.1%) in the 18–45 years age category. The majority of primary earners among patients’ families belong to the informal sector, and 50.6% had not received a formal education. With reference to Pakistan’s national household consumption quintiles, 46.8% of the participants belonged to the poorest quintile, while 12.6% were from the wealthiest quintile. Patient characteristics are presented in Table 2.

**Table 2:** Socioeconomic characteristics.


CHARACTERISTIC	ALL PROCEDURES (n = 526)	C-SECTION (n = 241)	LAPAROTOMY (n = 143)	ORIF (n = 142)

Age, No. (%)				

<18	58 (11.0)	1 (0.4)	24 (16.8)	33 (23.2)

18–45	395 (75.1)	239 (99.2)	85 (59.4)	71 (50.0)

>45	73 (13.9)	1 (0.4)	34 (23.8)	38 (26.8)

Sex, No. (%)				

Male	218 (41.4)	0 (0)	101 (70.6)	117 (82.4)

Female	308 (58.6)	241 (100)	42 (29.4)	25 (17.6)

Education, No. (%)				

None	201 (38.2)	91 (37.8)	59 (41.3)	51 (35.9)

Informal	65 (12.4)	37 (15.4)	16 (11.2)	12 (8.5)

Primary	102 (19.4)	39 (16.2)	25 (17.5)	38 (26.8)

Secondary	135 (25.7)	58 (24.1)	41 (28.7)	36 (25.4)

Graduate	23 (4.4)	16 (6.6)	2 (1.4)	5 (3.5)

Occupation of primary earners, No. (%)				

Informal sector	312 (66.4)	140 (63.1)	85 (66.4)	87 (72.5)

Self-employed	59 (12.6)	31 (14.0)	17 (13.3)	11 (9.2)

Private employee	52 (11.1)	29 (13.1)	11 (8.6)	12 (10.0)

Government employee	45 (9.6)	22 (9.9)	14 (10.9)	9 (7.5)

Unemployed	2 (0.4)	0 (0)	1 (0.8)	1 (0.8)

Per capita household consumption quintile, No. (%)				

1st	246 (46.8)	109 (45.2)	72 (50.4)	65 (45.8)

2nd	89 (16.9)	44 (18.3)	20 (14.0)	25 (17.6)

3rd	67 (12.7)	33 (13.7)	15 (10.5)	19 (13.4)

4th	58 (11.0)	31 (12.9)	16 (11.2)	11 (7.8)

5th	66 (12.6)	24 (10.0)	20 (14.0)	22 (15.5)

Household poverty, No. (%)				

World Bank	279 (53.0)	124 (51.5)	80 (55.9)	75 (52.8)

National	296 (56.3)	133 (55.2)	84 (58.7)	79 (55.6)

Province				

Sindh	171 (32.5)	78 (32.4)	49 (34.3)	44 (31.0)

KPK	120 (22.8)	57 (23.7)	34 (23.8)	29 (20.4)

Punjab	114 (21.7)	45 (18.7)	33 (23.1)	36 (25.4)

Balochistan	80 (15.2)	40 (16.6)	20 (14.0)	20 (14.1)

ICT	41 (7.8)	21 (8.7)	7 (4.9)	13 (9.2)


### Access and financial indicators

It was determined that 73.8% of patients had 2-hour access to the facility where they underwent their respective surgical procedures. The proportion of patients with 2-hour access was higher for C-section patients (81.7%) as compared to laparotomy patients and patients who underwent surgical management of a fracture (69.2% and 64.8%, respectively). Access data are listed in Table 3.

**Table 3:** Geographic access.


	ALL PROCEDURES (n = 526)	C-SECTION (n = 241)	LAPAROTOMY (n = 143)	ORIF (n = 142)

Location of residence in reference to hospital				

Same city	267 (50.8)	144 (59.8)	66 (46.2)	57 (40.1)

Other city	259 (49.2)	97 (40.3)	77 (53.8)	85 (59.9)

2-hour access	388 (73.76)	197 (81.7)	99 (69.2)	92 (64.8)


A majority (53%) of patients were poor at baseline with proportions ranging from 51.5% for C-section patients to 55.9% for laparotomy patients. Among the 247 patients who were categorized as non-poor at baseline (i.e. before the surgical need arose), 91 (36.8%) experienced IHE (Table 4). The overall proportion of patients with IHE was 70.3%, with the proportion ranging from 68.1% for C-section to 72.7% for laparotomy patients. The proportion of patients who experienced CHE was 79.5%, with the highest proportion seen in patients undergoing surgical management of a fracture (85.9%) and the lowest in C-section patients (74.7%).

**Table 4:** New impoverishment among non-poor patients.


NON-POOR PATIENT GROUPS	NEW IMPOVERISHMENT, NO. (%)

Overall (n = 247)	91 (36.8%)

C-section (n = 117)	40 (34.2%)

Laparotomy (n = 63)	24 (38.1%)

ORIF (n = 67)	27 (40.3%)


### Direct medical and non-medical costs

The median total direct costs expended by the patients were USD 338.5 (IQR 495.5), which included direct medical costs of USD 136.6 (IQR 297.3) and direct non-medical costs of USD 162.8 (IQR 254.1). Among the three procedures, the greatest cost was incurred for surgical management of a fracture at US 513.5 (756.8). Hardship financing was experienced by 50.95% of all patients; the greatest impact was seen in patients undergoing surgical management of a fracture, with 55.63% utilizing hardship financing. CHE, IHE, and hardship financing data are presented in Table 5.

**Table 5:** Financial risk protection.


	ALL PROCEDURES (n = 526)	C-SECTION (n = 241)	LAPAROTOMY (n = 143)	ORIF (n = 142)

Financial indicators				

Catastrophic health expenditure	418 (79.5)	180 (74.7)	116 (81.1)	122 (85.9)

Impoverishing health expenditure	370 (70.3)	164 (68.1)	104 (72.7)	102 (71.8)

Direct costs, median (IQR)				

Medical	136.6 (297.3)	117.6 (213.5)	148.9 (337.8)	168.9 (507.6)

Nonmedical	162.8 (254.1)	118.9 (191.9)	170.3 (237.8)	273.6 (389.2)

Total	338.5 (495.5)	251.4 (330.3)	365.1 (563.5)	513.5 (756.8)

Hardship financing experienced	268 (51.0)	117 (48.6)	72 (50.4)	79 (55.6)


## Discussion

This is the first systematic national survey to document access and financial protection indicators for surgical patients in Pakistan. We found that a substantial number of patients face long travel times to access essential care, and a high percentage of patients experience IHE and CHE during this process.

The most common surgical procedure in our patients was C-section, and this number was limited by our cap of any single procedure not exceeding 50% of the patients interviewed. Our non-C-section patients were predominantly male, low-educated, poor, and between the ages of 18–45 years, which is reflective of the segment of the Pakistani population reliant on public sector hospitals for care. This demographic also aligns fairly well with data from neighboring countries [18]. However it should be noted that direct comparisons are difficult because of different settings, time frames in which data was collected, and different inclusion and exclusion criteria between studies [18,19]. The gender disparity in accessing surgical care is a source of concern. Similar to our findings, previous studies from Pakistan have demonstrated a male preponderance in patients presenting with acute abdomen injuries [18,19]. An Indian [20] and Afghan [21] cohort had a male predominance (58% in both studies) in patients with acute abdomen presenting to tertiary care facilities. This gender disparity is more marked in our cohort, where 70% of all patients who underwent a laparotomy were male. A study from rural Northern Pakistan showed that the prevalence of acute abdomen increased with the age of the patient [18,19]. In contrast, our patient cohort was predominantly in the 18–45 years category.

These comparisons between the demographics of surgical patients indicate that the findings of our study are not unique to the Pakistani setting. Further research is needed to explore whether the male predisposition for acute abdomen and the stark differences in age at presentation with an acute abdomen is due to biological differences or due to certain segments of the population (e.g., women and the elderly) facing additional hindrances in the ability to access care.

A well-known way to assess different delays faced by patients seeking care is the three-delays model. The first delay is due to the time taken by patients or their family members to decide to seek appropriate care; the second delay happens when seeking transportation and trying to physically access a facility; while the third delay occurs when patients have reached an appropriate facility but are required to wait to receive the necessary care [22]. Our study and others [19] show that Pakistanis face any combination of these delays when seeking care. This is reflected in the access indicator in our study, where 26% of patients reported a travel time of more than 2 hours to reach the healthcare facility.

Studies from Afghanistan [21] and India [20] showed similar results of delays experienced before reaching a hospital with adequate surgical facilities. In the Indian study, two out of three patients only reached a medical facility with the capacity to treat an acute abdomen more than 24 hours after symptom onset. Predominantly because patients tended to seek care closer to home from facilities, providers were unable to identify the true extent of the disease or because services weren’t available [20]. Similarly, a 2002 study from rural Northern Pakistan showed that a majority of patients sought initial care close to home before eventually reaching the first level or specialized hospital, with eventually only 13% of patients accessing the necessary surgical care [18]. A study from Karachi mapped the geographical origins of patients who sought care at a tertiary hospital with an acute abdomen [23]. The results showed that 30% of the patients in their cohort came from outside of the city, some as far as Kashmir and KP. The researchers summarized that this is due to a lack of adequate surgical care closer to home, understaffing of surgeons at public hospitals, and the high out-of-pocket expenses incurred at private hospitals. Our study recorded an even higher number of patients (50%) seeking care from outside the city where the tertiary hospital is located. Such delays in accessing care are inevitably linked with increased morbidity and mortality on the one hand, and increased financial burden on the other [24]. It also affects efficiency at the tertiary care hospitals that receive a high volume of less complicated cases that could well be managed at first-level facilities closer to home [25,26,27]. Resources that should be used for specialized care services, research, and education are thus diverted to essential care provision. Facing multiple types of delays and having to seek care at different types of health facilities before being able to receive adequate care seems to be a critical challenge across the region that needs to be addressed systematically.

CHE affects poor people everywhere in the world and is not an issue unique to LMICs [28]. Certain factors predispose populations to be more affected by CHE. These factors include: lack of social security, including universal health coverage, availability of advanced medical services requiring out-of-pocket payment, and pre-existing poverty or inability to pay [28,29]. Pakistan’s health care system provides a perfect mix of these factors [30,31], explaining the high rates of CHE and IHE we identified in our cohort. For example, medicines, supplies, and laboratory tests are often not available at public sector hospitals and need to be paid for by the patient’s family as an out-of-pocket expenditure [30]. The unavailability of consumables and services in the public sector is not unique to Pakistan: inefficient supply chain processes and poor healthcare governance is well documented in many low-and-middle-income countries [23]. A large portion of direct expenditures consists of non-medical expenses, such as transportation and accommodation, which are inevitably higher when patients must travel long distances and spend prolonged time away from home. Unsurprisingly, this results in a significant proportion of non-poor patients becoming newly impoverished, as evidenced by our data.

Shrime and colleagues used economic modeling to determine the specific risk of CHE when seeking surgical care and found that countries in sub-Saharan Africa and South Asia were at greatest risk [6]. This is in stark contrast with the risk of CHE for all health conditions which is more evenly spread around the globe [28]. These results seem to indicate that, as compared to other types of medical care, surgical care has not been integrated into social security and health insurance packages. Additionally, based on multi-country comparisons, two factors have been identified to be responsible for driving the risk of CHE in case of surgery upwards: reliance of the health care system on outside funding and a smaller percentage of the GDP dedicated to health [6]. Pakistan is known to spend a notoriously low percentage of its GDP on health, about 1.5%, which is well below the World Health Organization’s (WHO) recommended minimum of 5% and significantly less than other LMICs [3]. For Pakistan specifically, authors have estimated that 75.2% of the Pakistani population was at risk of CHE if immediate surgery was needed, with this percentage ranging from 46.2% to 96.2% among the richest and poorest quintiles respectively [6]. Our results are well aligned with these estimations for Pakistan: we found a slightly higher overall risk of CHE at 79% for the cohort and a markedly high 85% risk of CHE for patients in need of fracture repair. It is important to note that while previous estimates for the financial impact of surgery in Pakistan were mainly based on modeling techniques, our study is the first to quantify financial impact based on primary, nationwide data.

Much less data is available in literature about the risk of IHE after seeking surgical care. A study from Sierra Leone found that people from the lowest and second lowest income quintile have a risk of 85% and 75% respectively of experiencing IHE after a C-section, compared to only a 10% risk of IHE in the highest income quintile. Additionally, this study found that uneducated people and people with lower incomes spend more on transport, food and accommodation when seeking care compared to the richer quintiles, inevitably leading to a higher risk of IHE. In our cohort, 34% of women were newly impoverished and 68% of women experienced IHE and were living under the poverty line after their C-section [32]. These numbers suggest that public tertiary hospitals in Pakistan cater for patients of the lower income quintiles, but most likely the poorest of the poor do not make it to tertiary hospitals and seek care elsewhere at a hospital or clinic closer to home, or not at all. A focus on strengthening surgical services at the first level could improve access significantly.

Several limitations are present in the design and conduct of this study. First, only patients who received surgical care were included in the survey. Patients who never sought care or who were unable to receive a procedure were not included. Second, the survey only captures costs before and during the operative period. All costs incurred post-operatively or for long-term care or rehabilitation were not assessed. Consequently, our results may be an underestimate of the financial burden of surgical care in Pakistan. Only patients seeking care at a public tertiary care hospital were included in this study, which limits the generalizability of our results to the entire population since a majority of Pakistanis access care in the private sector. We believe more accurate assessments of surgical systems are only possible by integrating relevant questions in the large-scale household survey conducted by local and international organizations.

Despite the limitations, this study provides valuable baseline data to health policymakers for reform efforts that are underway. Strengthening surgical infrastructure and services at the existing network of public sector first-level facilities have the potential to dramatically improve emergency and essential surgical care across the country. Availability of care closer to home would improve accessibility for high-burden procedures and minimize delays, and at the same time reduce the financial burden of transportation outside of urban areas. Pakistan is the first country in South Asia to embark on a national surgical system strengthening effort. The government launched the National Vision for Surgical Care 2020–25 in July 2021, with concrete plans to implement an Essential Package of Health Services, which has a strong component of surgical care. These are vital steps towards universal health coverage, of which surgical care is an integral component, to increase access and decrease the risk of CHE for patients when seeking surgical care in Pakistan. We believe that this study can serve as a blueprint for further studies in the region, and beyond and can serve as guidance for further research. The impact of the planned Universal Health Coverage (UHC) package on the accessibility and affordability of surgical care in Pakistan will have to be further explored, evaluated, and, if necessary, further addressed through policy changes. The inevitable dynamic between local beliefs, culture, and health- seeking behavior for surgical care deserves further attention as well, and will be necessary to understand better if we want to truly provide access to care for every Pakistani by 2030. Last but not least, understanding how patients interact with the health care system before reaching a facility is paramount to better understanding the needs and gaps at the secondary level.

## Additional File

The additional file for this article can be found as follows:

10.5334/aogh.3784.s1 Supplemental Material. Patient Experience Pakistan Lancet Indicators Modified.

## References

[1] Central Intelligence Agency. Pakistan. The World Factbook. https://www.cia.gov/the-worldfactbook/countries/pakistan/. Published 2022.

[2] Zaidi SA, Bigdeli M, Langlois EV, et al. Health systems changes after decentralization: progress, challenges and dynamics in Pakistan. BMJ Glob Health. 2019; 4(1): e001013. DOI: 10.1136/bmjgh-2018-001013 PMC6357909 30805206

[3] World Health Organization. Pakistan Health Financing System Review 2019. Geneva; 2019. https://phkh.nhsrc.pk/sites/default/files/2021-07/Pakistan_Health_Financing_System_Review_WHO_2019.pdf.

[4] Meara JG, Leather AJ, Hagander L, et al. Global Surgery 2030: evidence and solutions for achieving health, welfare, and economic development. Lancet. 2015; 386(9993): 569–624. DOI: 10.1016/S0140-6736(15)60160-X 25924834

[5] Farmer PE, Kim JY. Surgery and global health: a view from beyond the OR. World J Surg. 2008; 32(4): 533–536. DOI: 10.1007/s00268-008-9525-9 18311574 PMC2267857

[6] Shrime MG, Dare AJ, Alkire BC, et al. Catastrophic expenditure to pay for surgery worldwide: a modeling study. Lancet Glob Health. 2015; 3(Suppl 2): S38–44. DOI: 10.1016/S2214-109X(15)70085-9 25926319 PMC4428601

[7] Anderson GA, Ilcisin L, Kayima P, et al. Out-of-pocket payment for surgery in Uganda: The rate of impoverishing and catastrophic expenditure at a government hospital. PLoS ONE. 2017; 12(10): e0187293. DOI: 10.1371/journal.pone.0187293 29088302 PMC5663485

[8] National Surgical Planning Resources | pgssc. Accessed March 18, 2022. https://www.pgssc.org/national-surgical-planning.

[9] Holmer H, Bekele A, Hagander L, et al. Evaluating the collection, comparability and findings of six global surgery indicators. Br J Surg. 2019; 106(2): e138–e150. DOI: 10.1002/bjs.11061 30570764 PMC6790969

[10] PPP conversion factor, private consumption (LCU per international $) – Pakistan | Data. Accessed March 18, 2022. https://data.worldbank.org/indicator/PA.NUS.PRVT.PP?locations=PK.

[11] Poverty gap at $3.20 a day (2011 PPP) (%) | Data. Accessed March 18, 2022. https://data.worldbank.org/indicator/SI.POV.LMIC.GP.

[12] Towards A Stable Economy And Politics – PIDE. Accessed March 18, 2022. https://pide.org.pk/research/towards-a-stable-economy-and-politics/.

[13] Risk of impoverishing expenditure for surgical care (% of people at risk) | Data. Accessed March 18, 2022. https://data.worldbank.org/indicator/SH.SGR.IRSK.ZS.

[14] Risk of catastrophic expenditure for surgical care (% of people at risk) | Data. Accessed March 18, 2022. https://data.worldbank.org/indicator/SH.SGR.CRSK.ZS.

[15] Harris PA, Taylor R, Thielke R, et al. Research electronic data capture (REDCap)—a metadata-driven methodology and workflow process for providing translational research informatics support. J Biomed Inform. 2009; 42(2): 377–381. DOI: 10.1016/j.jbi.2008.08.010 18929686 PMC2700030

[16] Harris PA, Taylor R, Minor BL, et al. The REDCap consortium: Building an international community of software platform partners. J Biomed Inform. 2019; 95: 103208. DOI: 10.1016/j.jbi.2019.103208 31078660 PMC7254481

[17] Stata | FAQ: Citing Stata software, documentation, and FAQs. Accessed March 18, 2022. https://www.stata.com/support/faqs/resources/citing-software-documentation-faqs/.

[18] Ahmed M, Shah M, Luby S, et al. Survey of surgical emergencies in a rural population in the Northern Areas of Pakistan. Trop Med Int Health. 1999; 4(12): 846–857. DOI: 10.1046/j.1365-3156.1999.00490.x 10632993

[19] Samad L, Jawed F, Sajun SZ, et al. Barriers to accessing surgical care: a cross-sectional survey conducted at a tertiary care hospital in Karachi, Pakistan. World J Surg. 2013; 37(10): 2313–2321. DOI: 10.1007/s00268-013-2129-z 23765083

[20] Khanapure S, Nagral S, Nanavati AJ. A study of events between the onset of symptoms and hospital admission in patients with acute abdomen. Natl Med J India. 2017; 30(2): 65–68. 28816211

[21] Danish A. A retrospective case series study for acute abdomen in general surgery ward of Aliabad Teaching Hospital. Ann Med Surg (Lond). 2022; 73: 103199. DOI: 10.1016/j.amsu.2021.103199 35003728 PMC8717441

[22] Barnes-Josiah D, Myntti C, Augustin A. The ‘three delays’ as a framework for examining maternal mortality in Haiti. Soc Sci Med. 1998; 46(8): 981–993. DOI: 10.1016/S0277-9536(97)10018-1 9579750

[23] Khan MS, Haider SA, Ashfaq A, et al. Geospatial mapping of patients presenting for Emergency Laparotomy to a Private Sector Tertiary Care Hospital in Pakistan. J Pak Med Assoc. 2019; 69(Suppl 1): S37–S40. 30697017

[24] Shah N, Hossain N, Shoaib R, et al. Socio-demographic characteristics and the three delays of maternal mortality. J Coll Physicians Surg Pak. 2009; 19(2): 95–98. DOI: https://doi.org/02.2009/JCPSP.9598 19208312

[25] Sanchos J, Sequeira P. Health Facility Assessment Pakistan National Report. Lahore; 2012. https://docplayer.net/25154130-Health-facilityassessment-pakistan-nationalreport-trf-technical-resourcefacility.html.

[26] Ihsan A, Muhammad N, Fatima B, et al. Need Assessment of Trauma Centres in Punjab. Punjab Economic Research Institute; 2018.

[27] Irfan FB, Irfan BB, Spiegel DA. Barriers to accessing surgical care in Pakistan: healthcare barrier model and quantitative systematic review. J Surg Res. 2012; 176(1): 84–94. DOI: 10.1016/j.jss.2011.07.046 22079839

[28] Xu K, Evans DB, Kawabata K, et al. Household catastrophic health expenditure: a multicountry analysis. Lancet. 2003; 362(9378): 111–117. DOI: 10.1016/S0140-6736(03)13861-5 12867110

[29] Ezat Wan Puteh S, Almualm Y. Catastrophic Health Expenditure among Developing Countries. Health Syst Policy Res. 2017; 04(01). DOI: 10.21767/2254-9137.100069

[30] Issac A, Chatterjee S, Srivastava A, et al. Out of pocket expenditure to deliver at public health facilities in India: a cross sectional analysis. Reprod Health. 2016; 13(1): 99. DOI: 10.1186/s12978-016-0221-1 27557904 PMC4997742

[31] Social Protection in Pakistan. Accessed March 18, 2022. https://www.worldbank.org/en/country/pakistan/brief/social-protection-in-pakistan.

[32] van Duinen AJ, Westendorp J, Ashley T, et al. Catastrophic expenditure and impoverishment after cesarean section in Sierra Leone: An evaluation of the free health care initiative. PLoS ONE. 2021; 16(10): e0258532. DOI: 10.1371/journal.pone.0258532 34653191 PMC8519447

