# Supplementary material for: Access and Financial Burden for Patients Seeking Essential Surgical Care in Pakistan
Source: Ann Glob Health. 2022 Dec 20;88(1):107. doi: 10.5334/aogh.3784 (PMC9784127; doi:10.5334/aogh.3784)
Supplement: Supplemental Material. — Patient Experience Pakistan Lancet Indicators Modified. [file agh-88-1-3784-s1.pdf]

PATIENT EXPERIENCE
PAKISTAN LANCET INDICATORS MODIFIED

Under the bold items the questions to be asked.

Paragraphs in the boxes are explanations during the interview for the respondent. Read these out loud and be sure that the person understands the explanation.

I- Registration

1. **Record ID:**
2. **Data entered by:**
3. **Date of interview:**

II- Residential Address:

1. **City/Village:**
2. **Area:**
3. **Colony/Town:**
4. **House/Street number:**
5. **Any important place nearby:**
6. **Hospital name:**
7. **Block code:**

Assalam-alaikum, my name is __________ and I work for the Global Health Directorate - Indus Health Network. We are trying to document the experience of patient who is seeking surgical care and the costs associated with this surgical care. Surgical care includes care delivered by a surgeon, a medical doctor who cures patients by taking care of wounds and broken bones or cutting out masses. Sometimes surgeons must put you to sleep to do these things, and other times they must only numb the hurt body part-

Procedure of the Study

To find out the experience of patient who is seeking surgical care and the costs associated with this surgical car we would like to ask you and questions. We will ask questions about your personal information, the time taken for you to reach this hospital, about the type of surgery that was performed and your satisfaction with the service you received. We will also ask your household resources and income status and the expenditures incurred during this hospital stay.

This survey will take about 30 min to 1 hour. You have the right to not participate in the survey, or to stop during the interview.

We will ensure that all information collected from you will only be accessible to the Indus Health Network research team and stored in a password protected, secure location. All other data collected from you will be kept for 5 years in the records and then destroyed as per the hospital's policy.

Benefits of the Study

By asking these questions, we hope to involve specific institutions to improve the difficulties faced by patients seeking surgical care.

Risks associated with the study

There are no risks associated with being part of the study and it will only take some of your valuable time.

Do you have any other questions about the study?

III- Informed consent:

1. Would you like to participate in this survey?

   ___ Yes
   ___ No

2. If no, what is the reason? _____________
   a. Shortage of time
   b. No willingness
   c. No reason
   d. Not beneficial
   e. Other reason: explain….

[Without informed consent you cannot proceed. Make sure the person understands the purpose of this survey. If they don't want to participate, ask why and mark this.]

IV- DEMOGRAPHICS

1. **Record ID:**
2. **Data entered by:**
3. **Sex**
    a. **Male**
    b. **Female**
4. **Age**
5. Have you ever attended school?
    a. Yes
    b. No
6. If no, what is the reason? _____________
    a. Shortage of time
    b. No willingness
    c. No reason
    d. Not beneficial
    e. Other reason:
    f. Explain….
7. Any informal education:
    a. Madrassa
    b. Adult literacy
    c. Home schooling
    d. Self learnt (reading)
    e. Self learnt (reading and writing)
8. What is the highest educational level that you have achieved or are currently following?
    a. None (includes nursery)
    b. Primary school
    c. Secondary school (matriculation)
    d. Higher Secondary school (intermediate)
    e. Tertiary (Bachelors)
    f. Graduate degree (Master degree, PhD)

9. Are you able to read and write in any language?
   [For adults and children who are currently learning how to read and write answer: 'No']
    a. Yes
    b. No
10. What is your primary occupation?
    a. Unemployed
    b. Home Maker
    c. Domestic Helpers
    d. Farmers
    e. Self Employed-Own business
    f. Govt Service Employees
    g. Private Service
    h. Labour
    i. Other
11. Please explain any other profession

_

12. **Tribe**
    What is your ethnic background? *[In case of refusal to answer, ask the person his place of birth]*
    a. Muhajir
    b. Punjabi
    c. Pakhtoon
    d. Sindhi
    e. Memon
    f. Seraiki
    g. Bihari
    h. Balochi
    i. Gujrati
    j. Brohi
    k. Hindko
    l. Bengali
    m. Jadgal
    n. Kathiawari
    o. Other
    p. Don't know
13. Please write if any other
14. Do you stay healthy usually?
    a. Yes
    b. No
15. During the last year, how many weeks were you sick?
    _____ weeks
16. During the last year, how many times did you visit clinic/hospital or doctor/nurse for consultation? _____
17. Have you recovered from your illness?
    a. Yes
    b. No

V- Surgery, Choice of Hospital and Patient Experience

1. **Record ID:**
2. **Data entered by:**

SURGERY

3. **What type of surgery did you have?**
    a. Laparotomy
    b. Cesarean section
    c. Fixing operation with surgeryixation of Open fracture repair

4. **What was the date of surgery?**
    _____ Day _____ Month _____ Year

5. **Was this surgery anticipated?**
    a. Yes
    b. No

REASON FOR CHOICE OF HOSPITAL

6. Why did you choose the hospital (distant or near)?
    a. Near to my house
    b. Best doctors, nurses, other staff in this area
    c. Could afford it
    d. Medicines are available

e. I heard it had a quality improvement project
f. friend or relative has used it before and recommended it
g. I have used it before and had a good experience
h. Told to come here from other facility after problem started (referred)
i. Any Other (please specify): _________________
j. Don't know
k. No response

PATIENT EXPERIENCE QUESTIONS

The length of time you waited before you were seen.

Less than an hour	1 hour	1-2 hours	2-3 hours	More than 3 hours

How satisfied are you that the doctor listened to you carefully?

Not satisfied	Somewhat satisfied	Satisfied	Very satisfied	Extremely satisfied

How satisfied are you with doctor's medical knowledge and skills?

Not satisfied	Somewhat satisfied	Satisfied	Very satisfied	Extremely satisfied

How satisfied are you with the level of respect the doctor showed you?

Not satisfied	Somewhat satisfied	Satisfied	Very satisfied	Extremely satisfied

How satisfied are you with the amount of time the doctor spent with you in the visit?

Not satisfied	Somewhat satisfied	Satisfied	Very satisfied	Extremely satisfied

Overall, thinking about your entire visit, how satisfied were you with the care you received?

Not satisfied	Somewhat satisfied	Satisfied	Very satisfied	Extremely satisfied

VI- TRANSPORTATION

1. **Record ID:**
2. **Data entered by:**
3. **What was the first symptoms of your illness?**
   ____
4. **How much time did you take to decide to go to the hospital after the first symptom?**
   ____
5. **How many transports you used to get to the hospital?**
   a. **One ________**
   b. **Two ________**
   c. **Three _______**
6. **What was the first transport you used to get to the hospital?**
   a. Ambulance
   b. Public transport (bus)
   c. Taxi
   d. Rickshaw
   e. Own Car

f. Motorcycle
g. Bicycle
h. Boat
i. Animal
j. On foot
k. Carried

7. What was the second transport you used to get to the hospital?

a. Ambulance
b. Public transport (bus)
c. Taxi
d. Rickshaw
e. Own Car
f. Motorcycle
g. Bicycle
h. Boat
i. Animal
j. On foot
k. Carried

8. What was the third transport you used to get to the hospital?

a. Ambulance
b. Public transport (bus)
c. Taxi
d. Rickshaw
e. Own Car
f. Motorcycle
g. Bicycle
h. Boat
i. Animal
j. On foot
k. Carried

9. How long did you have to wait for the arrangement of transportation to get to this health facility? (hours)

10. How long did it take you to get to the hospital after getting the transport?
____

11. How much did you or your family spend on transportation to reach this health facility (in PKR)? _____________

12. Do you always have the above amount with you?

a. Yes
b. No

13. How long you had to wait to be seen by the doctor after reaching the hospital?

VII- FINANCIAL RISK PROTECTION SURVEY

1. **Record ID:**
2. **Data entered by:**

Typical Monthly Income

3. Date of interview:
4. Person being interviewed:
    a. Patient
    b. Attendant
    c. Both
5. What is the size of your household, including yourself (how many members normally live in your house)?
6. What occupation is held by the person who makes the most money in your household *(primary breadwinner?)?*
7. How much does your household earn in an average month?

Typical Monthly Expenditure

8. How much money does your household spend on food to eat, water, juice, or cold drink (soda) to drink?
9. Do you always have the above amount with you?How much money does your household spend on livestock (including cows, goats, sheep, chickens etc.) every month?
10. Excluding this hospital course, how much money does your household usually spend on health care, including medicines, fees for doctors or hospital visits, fees for traditional healers?
11. How much money does your household spend on transport?
12. How much rent does your household pay for your house?
13. How much money does your household spend on other household matters such as clothes, improvements, and decoration of your house etc?
14. How much money does your household spend on education per year for all the children you support, including school fees and books?
15. Do you have other monthly expenditures?
16. If so, how much are these other expenditures?

Hospitalization Associated Expenditures

17. Did your household have to borrow money to pay for this hospitalization?

    Yes _______

    No____

18. If yes, how much?

19. Did your household have to sell any land or possessions (including livestock) to pay for this hospitalization?

    Yes______

    No_______

20. If yes, then how much you sold it for?

21. Did your household have to permanently stop sending any children to school, or did you pay reduced school fees to pay for this hospitalization?

    Yes_____

    No______

22. How much did your household have to spend on food during this hospitalization?

23. How much in daily wages do you feel your household has lost due to this hospitalization?

24. Did you or anyone in your household permanently lose a job because of this hospitalization?

25. How much did you receive in outside funds from charity or from the social worker/hospital fund to pay for your hospitalization for all goods and services?
